# Measuring age-specific variations in income-related inequalities in smoking behavior in Germany

**DOI:** 10.1080/21642850.2014.891946

**Published:** 2014-03-31

**Authors:** Martin Siegel

**Affiliations:** ^a^ Department of Health Care Management, Technische Universität Berlin, H80, Straße des 17. Juni 135, 10623Berlin, Germany; ^b^ Institute of Health Economics and Clinical Epidemiology, University Hospital of Cologne, Cologne, Germany

**Keywords:** concentration index, age-specific variations, income-related inequality, smoking behavior, Germany

## Abstract

Although monitoring smoking behavior is considered as most important to tackle the smoking epidemic, empirical evidence concerning age-specific variations of its income-related inequalities still seems scarce. This paper uses a semiparametric extension of the concentration index to measure age-specific variations of income-related inequalities in smoking behavior. First, current smoking is used to describe peoples’ actual smoking status. Second, ever-smoking is included to approximate how inequalities in smoking behavior changed with the evolution of the smoking epidemic. Finally, smoking cessation is considered to indicate an individual's ability to conquer the habit. Cross-sectional data from the 2009 survey of the German microcensus reveal that current smoking is most prevalent among adolescents and young adults, more common among the worse-off in younger age groups and concentrated among the better-off in older age groups. Concentration of ever-smoking among the economically deprived is only found for younger adults. Smoking cessation is more common among higher income ever-smokers in all age groups. One may deduce from these results that anti-smoking policies should particularly aim at younger individuals in lower-income households.

## 1. Introduction

A broad body of literature provides evidence on the various hazardous effects of smoking. For example, smoking increases the risk of pulmonary and cardiovascular diseases (Kamholz, 2004), asthma among adolescents (Genuneit et al., 2006) and lung cancer (de Groot & Munden, 2012; Lee, Forey, & Coombs, 2012; Peto et al., 2000). It is associated with premature mortality (Balia & Jones, 2008; Mons, 2011) and lower quality of life (Slama, 2008). To quit smoking may prevent the incidence of smoking-related diseases even in later mid-life (Peto et al., 2000). Balia and Jones (2008) found socio-economic inequalities in smoking to be an important contributor to persisting health inequalities. According to Schaap and Kunst (2009), monitoring socio-economic inequalities in tobacco consumption to design distinct anti-smoking policies for specific vulnerable groups may be a promising approach when aiming an equitable distribution of health. In this context, identifying age groups with particularly strong socio-economic gradients in tobacco consumption may be of particular interest for policy makers when designing anti-smoking policies.

Several studies describe the evolution of the smoking epidemic since the early twentieth century and find similar patterns in most industrialized countries (Giskes et al., 2005; Graham, 1996; Schulze & Mons, 2006). Smoking was rather uncommon among women and prevalence rates first rose among higher educated men. While becoming more common among the less educated, the smoking prevalence declined among individuals with higher social status. The faster decline in the prevalence of smoking among men leveled the initial gender differences in smoking behavior over time (Graham, 1996).

Persisting social gradients in smoking behavior are well documented for most industrialized countries. Individuals with lower socio-economic status, in general, have higher consumption levels, start smoking earlier in life and are less likely to quit (Schaap & Kunst, 2009). Germany exhibits similar patterns in terms of prevalence and inequalities (Helmert & Buitkamp, 2004; Lampert & Burger, 2004; Lampert & Thamm, 2004), and may thus be considered as a good example to further analyze socio-economic gradients in smoking behavior and the evolution of the smoking epidemic.

To the best of my knowledge, this is the first paper to explore age-specific variations in income-related inequalities in smoking behavior. This paper adds to the literature by measuring age-specific concentration indices for three smoking-related variables from the German microcensus 2009. Current smoking is used to describe individuals’ actual smoking status. Additionally, ever-smoking (i.e. former or current smoking) is included to approximate potential cohort effects to reveal possible changes of income-related inequalities in the light of the smoking epidemic's evolution during the twentieth century. Smoking cessation is measured as former smoking among the ever-smokers to assess the distribution of individuals’ ability to conquer a bad habit. One may agree that the commonly used (homogeneous) concentration index would not reveal variations in the socio-economic gradient between age groups. This paper applies a varying inequality index recently introduced by Siegel and Mosler (2013), which is a semiparametric extension of the concentration index. Local estimation based on a non-parametric smoothing approach allows this index to vary with some metric variable, thus to highlight variations of the income-related gradient in tobacco consumption with age.

## 2. Materials and methods

### 2.1. Data

Data are taken from the 2009 wave of the German microcensus. The microcensus is a representative survey of the German population conducted by the Research Data Centers of the Federal Statistical Office and the Statistical Offices of the Federal States (Forschungsdatenzentren der Statistischen Ämter des Bundes und der Länder). Comprising approximately 1% of the German households, the microcensus is considered as the most representative survey available for Germany (Reeske, Spallek, & Razum, 2009; Schimpl-Neimanns & Herwig, 2011).

The present paper uses the scientific use file comprising a random subsample of approximately 70% (*n *= 489,349) of the German microcensus. Children younger than 15 (64,808) were not asked about smoking and 80,968 individuals over 15 did not respond to the smoking-related questions. The data include information about smoking behavior for 343,573 individuals (179,659 female and 163,914 male) aged 15 or older, of which 26,597 observations (12,763 male and 13,834 male) had to be removed because of missing information on household income. The final sample comprises 316,976 (151,151 male and 165,825 female) individuals. The data comprise inverse probability weights which were computed for certain groups of the sample with respect to the underlying population. The inverse share of individuals excluded from the sample was computed separately for each of these groups and multiplied with the given sample weights. These adjusted sample weights are used in the following analyses to guarantee a representative sample for the German population even after excluding observations.

### 2.2. Variables

Interviewees were asked whether they currently were frequent, occasional or non-smokers. Non-smokers were then asked whether they were former frequent or occasional smokers. As one may agree that the subjective distinction between frequent and occasional smoking is rather weak, individuals are only grouped into smokers and non-smokers. The first outcome variable is current smoking, the second outcome is ever-smoking. Ever-smokers are individuals who either currently smoke or formerly smoked. As a third outcome, smoking cessation is measured as former smoking among the ever-smokers.

The socio-economic status variable used here is net equivalent household income. Data on income comprise all possible sources (e.g. from labor, capital or pensions). To account for the household size, total income is adjusted using the modified OECD equivalence scale (van Doorslaer, Koolman, & Jones, 2004; Siegel & Mosler, 2013). One may consider the reliability of current income as a measure for the socio-economic position after retirement as problematic for some countries. The German pension system, however, is considered to be highly status preserving (Brockmann, Müller, & Helmert, 2009). Approximately 90% of the German population are covered by the public pension scheme where benefits depend largely on compulsory contributions until retirement (Boersch-Supan & Wilke, 2004). The relative socio-economic position within one's age group is therefore unlikely to change considerably with retirement (Siegel & Mosler, 2013). That said, one may agree that income is a suitable measure for the age-specific socio-economic status here.

### 2.3. Measuring age-specific inequalities

The concentration index *C* has become a common measure of income-related inequalities in health (van Doorslaer et al., 2004; Kakwani, Wagstaff, & van Doorslaer, 1997; O'Donnell, van Doorslaer, Wagstaff, & Lindelow, 2008; Wagstaff, 2005; Wagstaff, Paci, & van Doorslaer, 1991). *C* was derived from the concentration curve which plots the cumulative share of outcome *y* against the cumulative share of the population ranked by income. *C* measures twice the area between the line of equality and the concentration curve and is bounded in the (−1; 1) interval. *C* is positive (negative), if the outcome is concentrated among the rich (poor). Where no inequality is observed, the concentration curve coincides with the line of equality and *C* equals zero (O'Donnell et al., 2008; Wagstaff et al., 1991).

Following Siegel and Mosler (2013), the convenient regression approach (Kakwani et al., 1997) is combined with the varying coefficient model (Li, Huang, Li, & Fu, 2002) to obtain the concentration index as a smooth function of some regressor 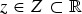,
(1) 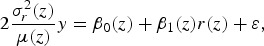

where 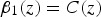. The *z*-specific mean of *y* is 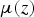, 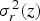 denotes the variance of the locally weighted fractional rank 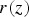 and 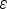 is the error term. Including the sample weights *w* and kernel weights 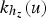 into the computation of 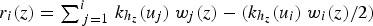 assures that its locally weighted mean and variance are 0.5 and 1/12 for any 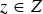. Note that the vector of sample weights *w* is rescaled for each *z* such that 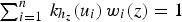 holds true for any 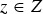 and individuals 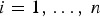 are sorted in ascending order by income.

Smoking prevalences may vary considerably across age groups and the bounds of concentration indices for binary variables depend inversely on the mean 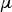, i.e. 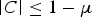 (Wagstaff, 2005, 2011). There is an ongoing discussion in the literature with a dissent on how to correct the concentration index (Carrieri & Wuebker, 2013; Erreygers, 2009; Kjellsson & Gerdtham, 2013; Wagstaff, 2005, 2011). The Wagstaff index *W* (Wagstaff, 2005, 2011) has been designed as an inequality indicator for binary variables. Wagstaff (2005, 2011) argues that the maximum possible inequality for a dichotomous health outcome is observed where the richest (poorest) 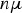 individuals accumulate all health (or ill-health). This paper adopts the Wagstaff index *W* such that 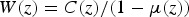, which rescales 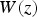 to a (−1; 1) interval irrespective of 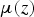. This choice intends to compensate variations in the concentration index caused by pure variations of the underlying prevalence, allowing comparisons of inequalities throughout the support of *z* (Siegel & Mosler, 2013).Equation 1, 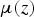 and 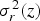 are estimated using a consistent Nadaraya–Watson estimator with kernel weights 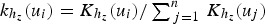 derived from a quartic kernel function and 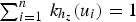. The quartic kernel assigns higher weights to observations closer to *z*, lower weights to observations further away from *z* and zero weight if an observation is outside the bandwidth. The bandwidth parameter 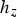 is chosen inversely to the local data density 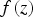 to include a wider range of *z* where data are scarce. Fan and Gijbels (1992) have shown that adaptive local smoothers generally yield good results and avoid the well-known boundary effect. See Siegel and Mosler (2013) for a more technical introduction of the varying inequality index and the computation of its confidence bands.

## 3. Results

The results in Table 1 for the full sample show that mean age is lower in the male than in the female sample. The average net equivalent household income is higher for men than for women in the full and the restricted sample. Women have a considerably lower prevalence of current and ever-smoking than men. The homogeneous Wagstaff indices demonstrate that current smoking is significantly concentrated among the worse-off for both sexes in the full and the (restricted) ever-smoker sample. Conversely, former smoking is concentrated among the better-off in all samples. Ever-smoking is significantly concentrated among individuals in lower income households in the male sample while no significant concentration is observed in the female sample. This is also reflected by the average income which is lower among the male ever-smokers compared with the full sample. According to Table 1, the prevalence of current smoking among the ever-smokers is higher for women than for men. Ever-smoking women are, on average, younger than ever-smoking men. The Wagstaff index for former smoking in Table 1 is exactly the negative of the Wagstaff index for current smoking in the ever-smoking subsample for both sexes. This is because ever-smokers can only be current or former smokers, and Wagstaff's corrected index always fulfills the so-called mirror condition (Erreygers, 2009; Wagstaff, 2011).

**Table 1.** Income inequality and income-related smoking inequality.

	Male			Female		
	Prevalence	*W*^a^	s.e.^b^	Prevalence	*W*^a^	s.e.^b^
*Full sample*	*n* = 151,151			*n* = 165,825		
Age	47.03^c^			48.93^c^		
Income	1597.32^c^	0.2896*^,^^d^	0.0046	1493.36^c^	0.2807*^,^^d^	0.0040
Ever-smokers	56.26%	−0.0834*	0.0071	37.36%	−0.0039	0.0059
Current smokers	31.33%	−0.1687*	0.0063	22.12%	−0.1108*	0.0065
Former smokers	24.93%	0.0843*	0.0067	15.24%	0.1408*	0.0073

*Ever-smokers*	*n *= 84,745			*n* = 60,131		
Age	48.64^c^			45.36^c^		
Income	1541.43^c^	0.2862*^,^^d^	0.0062	1493.60^c^	0.2850*^,^^d^	0.0069
Current smokers	55.69%	−0.1814*	0.0089	59.21%	−0.2027*	0.0101
Former smokers	44.31%	0.1814*	0.0095	40.79%	0.2027*	0.0121

Notes: Mean net equivalent household incomes and prevalences of current, ever and former smoking from the 2009 microcensus, Germany.

^a^Estimated income-related inequality (Wagstaff index *W*).

^b^Standard error.

^c^Mean income.

^d^Gini index (without Wagstaff's correction) for income.

*Significant at the 99% level.

**Figure 1.**
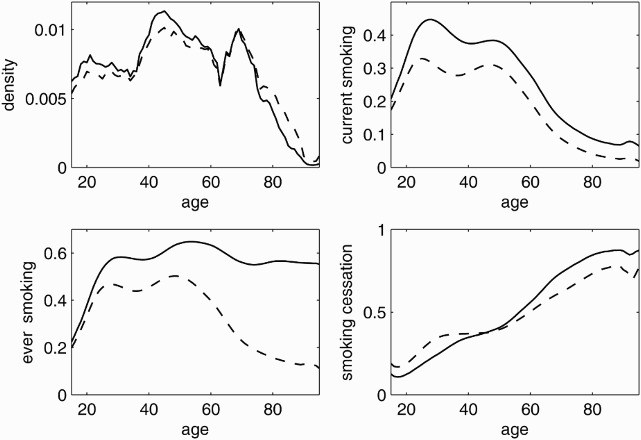
Age distribution and age-specific prevalence of smoking. Age-specific data density (upper left) and prevalence of current (upper right) and ever-smoking (bottom left) as well as smoking cessation among ever-smokers (bottom right) for men (solid) and women (dashed) from the 2009 microcensus, Germany.

The data density plot in Figure 1 presents the distribution of age in the male and female sample and corresponds to the population pyramid for Germany. The corresponding bandwidth parameter is highest for individuals older than 80 years and smallest for the 40–50 years old, and for women around 70 years of age. The age-specific prevalence of current smoking in Figure 1 shows similar patterns for men and women. The ever-smoking curve for females is similar to the current smoking curve but exhibits an approximately 10% higher level among adults. In contrast, the prevalence of ever-smoking differs from that of current smoking as it does not exhibit the decrease observed for current smoking among men older than 50 years old. The curves for smoking cessation for males and females intersect at age 46, suggesting that older female ever-smokers are less likely to have quit smoking than male ever-smokers.

**Figure 2.**
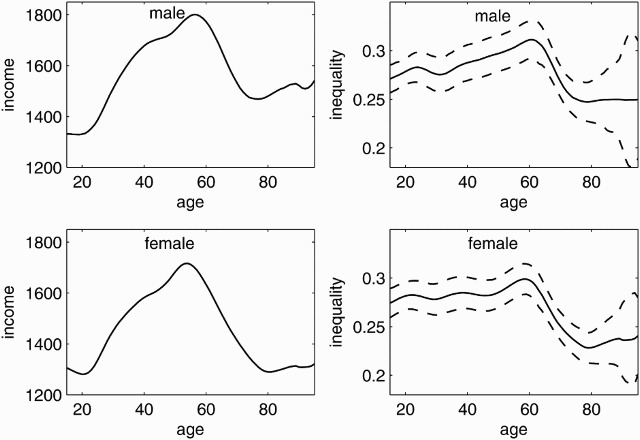
Age-specific mean income and income inequality. Age-specific mean incomes (solid lines, left graphs) and Gini indices (solid lines, right graphs) with 95% confidence intervals (dashed lines, right graphs) for men (top) and women (bottom) from the 2009 microcensus, Germany. Higher Gini indices indicate higher degrees of income inequality.

Figure 2 presents the age-specific mean and inequality of net equivalent household income for the unrestricted male and female samples. The results for age-specific mean income demonstrate that men have, on average, higher incomes in all age groups. The difference is most pronounced among the elderly over 70 years old. Income inequality is highest around the statutory retirement age of 65 years and lowest among the retired. Statistically significant income inequality persists among the elderly for both sexes.

**Figure 3.**
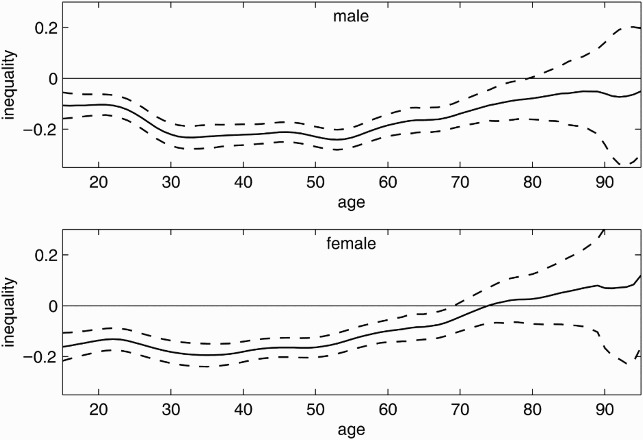
Age-specific inequality in current smoking. Age-specific inequality index (solid line) with 95% confidence intervals (dashed lines) for men (top) and women (bottom) from the 2009 microcensus, Germany. Negative values indicate concentration among the poor, positive values indicate concentration among the rich.

The varying Wagstaff indices in Figure 3 demonstrate the age-specific inequality of current smoking and suggest a concentration of current smoking among the worse-off. The index for men is negative for all age groups and significant for those younger than 80. In contrast, women exhibit an insignificant concentration among the better-off older than 74. The confidence bands for the indices for the male and female samples do not overlap for those aged between 52 and over 59 years old, suggesting a significantly stronger concentration of current smoking among lower incomes in the male compared with the female sample in this age group.

**Figure 4.**
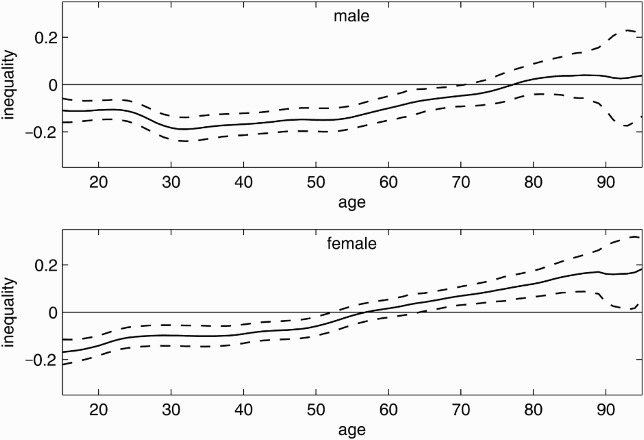
Age-specific inequality in ever-smoking. Age-specific inequality index (solid line) with 95% confidence intervals (dashed lines) for men (top) and women (bottom) from the 2009 microcensus, Germany. Negative values indicate concentration among the poor, positive values indicate concentration among the rich.

Figure 4 demonstrates the varying Wagstaff indices for ever-smoking. Similar to the graphs in Figure 3, the graphs in Figure 4 show a positive trend with increasing age for men over 30 and all women. Ever-smokers are (at the 5% level) significantly concentrated among the worse-off for males younger than 70 and females younger than 52. In contrast to the male sample, a significant concentration of ever-smokers among the higher incomes is observed for females older than 65 years old. One may read this as a cohort effect indicating that the risk of smoking ever in life shifted from the rich towards the poor during the twentieth century; the change was more pronounced in the female sample. The concentration of ever-smoking among lower-income adolescents is stronger in the female than in the male sample for those younger than 23 years old. For the older cohorts, women have a weaker concentration among the poor or, where the index is positive, a stronger concentration among the better-off than men. The concentration of ever-smokers in lower-income households is significantly stronger among males for those aged between 50 and 75 years old. Comparing the results in Figure 4 with those in Figure 3, one may note that the curves for current and ever-smoking follow similar patterns but at a somewhat higher level.

**Figure 5.**
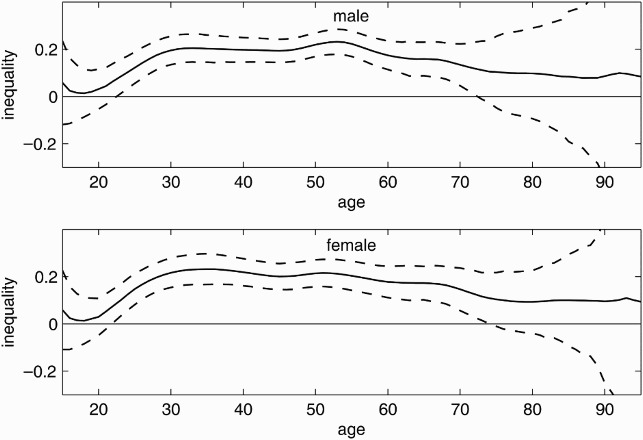
Age-specific inequality in smoking cessation among ever-smokers. Age-specific inequality index (solid line) for smoking cessation in the ever-smokers subsample with 95% confidence intervals (dashed lines) for men (top) and women (bottom) from the 2009 microcensus, Germany. Negative values indicate concentration among the poor, positive values indicate concentration among the rich.

Figure 5 presents the age-specific inequality indices for smoking cessation estimated from the restricted ever-smokers subsample. The graph suggests that higher income ever-smokers are more likely to quit smoking than those in lower income households. This is in line with the stronger concentration of current smoking compared with ever-smoking among the worse-off. The concentration among the better-off is significant at the 5% level for males between 23 and 72 years old and females between 22 and 74 years old. Comparing the varying Wagstaff indices for men and women yields no significant gender differences.

## 4. Discussion

The present paper applied a varying inequality index (Siegel & Mosler, 2013) to data from the 2009 survey of the German microcensus to describe age-specific income-related inequalities in smoking behavior. The income-related inequalities vary considerably with age, suggesting that a homogeneous index would neither have revealed the lower concentration of current and ever-smokers among adolescents in lower income households nor the pro-rich distribution of current and ever-smoking among the elderly. In contrast to Richter and Leppin (2007), a significant gradient related to household income for adolescents of both sexes was observed. Smoking cessation exhibits no significant income-related gradients for the youngest, which may be explained with two effects. First, the estimates for the varying Wagstaff indices are close to zero among the youngest. Second, only few stopped smoking in this group. It is important to mention here that such low prevalence rates increase the uncertainty by definition and hence widen the confidence bands.

Bauer, Göhlmann, and Sinning (2007) stress the importance of gender-specific policies to reduce smoking efficiently. The results exhibit an increasing gap between men and women in the prevalence of current and ever-smoking with increasing age (in other words decreasing for later birth cohorts). This is in keeping with the result that gender differences reduced during the twentieth century (Graham, 1996). For all age groups, however, men still exhibit a higher prevalence of current and ever-smoking than women. The age-specific inequalities of current and ever-smoking among the worse-off are similar for both sexes but significantly weaker among females aged 52–59 years old for current smoking and among females aged 50–75 years old for ever-smoking.

The results suggest that most males and females, if ever, start smoking in adolescence or early adulthood. Smoking prevalence is high among the younger adults and individuals apparently stop in mid-life. Sundmacher (2011) argues that smoking cessation is closely related to diagnoses of related diseases. One may speculate that such diseases rarely occur before mid-life and consider this as a possible explanation for both the current smoking and the smoking cessation curves. One may further speculate that the higher rates of current non-smoking among younger females compared with males may be related to pregnancies, as the average number of dependent infants is particularly high in households with 20–40-year-old females.

The income-related concentration of ever-smoking moved from the higher to the lower incomes during the twentieth century. Figure 4 suggests a change from pro-rich (positive index) to pro-poor (negative index) distributions with the male 1931 (age 78) and the female 1952 (age 57) birth cohorts. One may object measuring cohort effects in socio-economic gradients via household income and argue that income may vary over the life course while e.g. education could be considered as a durable asset. However, Schulze and Mons (2006) found similar results for the educational dimension of inequalities in smoking. They identify a change from the higher to the lower educated between 1921–1930 and 1931–1940 birth cohorts for men and between 1931–1940 and 1941–1950 birth cohorts for women. Comparing age-specific smoking prevalences for different educational levels in the underlying data yielded similar results.

The choice of the correction method for the concentration index may influence the results to some extent. There is an ongoing discussion about how to correct the concentration index *C* in the literature (Carrieri & Wuebker, 2013; Erreygers, 2009; Kjellsson & Gerdtham, 2013; Wagstaff, 2005, 2011). Kjellsson and Gerdtham (2013) argue that most rank-based inequality indicators measure how far a society is from a state of maximum inequality, where the major difference between such indicators is the definition of that state. The classical concentration index *C* (Kakwani et al., 1997; Wagstaff et al., 1991) considers a state where the richest (or poorest) person accumulates all health (or ill-health) as most unequal, and may thus be used as a relative inequality index for unbounded variables (an example is the Gini index for income). The index proposed by Erreygers (2009) considers a distribution where the better-off (worse-off) 50% have all health (or ill-health) as the most unequal scenario. This paper used the correction proposed by Wagstaff (2005), which considers a society where the poorest (richest) 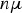 individuals accumulate all health (or ill-health) as the most unequal case. Kjellsson and Gerdtham (2013) give an extensive discussion about the properties and value judgments underlying the different indices.

Non-response to the voluntary smoking module is somewhat pro-poor in the data, i.e. lower income households were less likely to provide information about their smoking behavior. One may assume that the relation between income and smoking behavior also holds true for the non-respondents, and speculate that the income-related inequalities may be stronger than suggested by the results.

Germany has similar anti-smoking policies compared with other European Union member states, and previous studies found considerable similarities between Germany and most other industrialized countries in terms of smoking prevalence and inequalities. This is the first study using the varying inequality index to explore age-specific income-related inequalities in smoking behavior. One may speculate that similar age-specific patterns might be observable in other countries, however, one should be cautious with such generalizations.

The results presented in this paper demonstrate how the income-related gradients of smoking behavior vary between age groups. One may argue that these results are still descriptive, and that an age-specific decomposition analysis would be desirable to identify driving forces behind the observed results. Given the observed age-specific variations in income-related inequalities in smoking behavior, one may speculate that socio-economic characteristics such as income, education or employment status may have considerably different associations with an individual's smoking behavior at different ages. To the best of my knowledge, however, no varying coefficient models for nonlinear outcomes have been developed to date. Future research should involve the development, for example, of estimators for varying coefficient logistic or probit models to estimate age-specific variations in the determinants of health and health behavior, which would thereby facilitate age-specific decomposition analyses for binary outcome variables.

Analyses of health inequalities over the life course based on self-reported cross-sectional data may be subject to certain biases. It has, for instance, been shown that life expectancy is lower among the deprived. As smoking is related to severe diseases and premature mortality (Balia & Jones, 2008; Genuneit et al., 2006; Kamholz, 2004; Peto et al., 2000; Slama, 2008), smoking-related mortality may be considered as a possible confounder. Comparing the results with the overall mortality rates from the Human Mortality Database (2013), one may agree that mortality is unlikely to bias the results considerably before the age of 70 years. Another issue may be a potential bias through bad health selection into early retirement. However, this should lead to opposite results at least for ever-smokers. One would expect losses of incomes owing to bad health selection of ever-smokers into early retirement to accumulate them among the worse-off, which would lead to a concentration of smoking among them. Current and ever-smoking are, however, pro rich for the oldest. The measure of smoking cessation does not only include those who stopped smoking at the particular age. Although possibly overestimating the age-specific cessation rates, it may still work as an indicator reflecting the ability, say, to conquer a bad habit. Self-reported data on tobacco consumption may further be subject to underreporting because of social desirability adjusted responses. One who speculates that social desirability may be of higher importance among individuals in better-off households may argue that the results presented in this study potentially overestimate the socio-economic gradients in smoking behavior. To my knowledge, however, there is no evidence on whether socio-economic gradients in over- or underreporting of tobacco consumption exist in the underlying data, and potential influences on the results would be speculative.

## 5. Conclusions

This paper contributes to the literature by measuring age-specific income-related concentration indices of current smoking, ever-smoking and former smoking among ever-smokers. A significant concentration of current smoking and ever-smoking among the worse-off was found for adolescents and young adults. Those who quit smoking are concentrated among the better-off ever-smokers. The results suggest that anti-smoking policies should aim at adolescents and young adults in lower-income households.

The results support the common descriptions of the smoking epidemic (Giskes et al., 2005; Graham, 1996; Schulze & Mons, 2006), which first started among the better-off and, say, moved towards the deprived during the twentieth century. The smoking epidemic apparently proceeds similarly for males and females, but with some delay for the latter.
